# Effects of Dietary Pomegranate Syrup Supplementation on Growth Performance, Carcass Traits, Meat Quality, Oxidative Stress, and *GPx7* Gene Expression in Japanese Quail (*Coturnix coturnix japonica*)

**DOI:** 10.3390/vetsci13070708

**Published:** 2026-07-19

**Authors:** Aydin Daş, Besime Doğan Daş, Mücahit Kahraman, Mehmet Avci, Akın Yiğin, Sadik Serkan Aydin, Gülüzar Şengül, Oğuz Ağyar, Ahmet Yusuf Şengül

**Affiliations:** 1 Department of Animal Husbandry, Faculty of Veterinary Medicine, Harran University, 63200 Sanliurfa, Turkey; 2 Department of Animal Nutrition and Nutritional Disease, Faculty of Veterinary Medicine, Harran University, 63200 Sanliurfa, Turkey; 3 Department of Zootechnics and Animal Nutrition, Faculty of Veterinary Medicine, Bingöl University, 12000 Bingol, Turkey; 4 Department of Veterinary, Vocational School of Kahta, Adıyaman University, 02400 Adiyaman, Turkey; 5 Department of Animal Science, Faculty of Agriculture, Bingol University, 12000 Bingol, Turkey

**Keywords:** *GPx7* gene, oxidative stress, performance, pomegranate syrup, quail

## Abstract

The aim of this study was to evaluate the effects of pomegranate syrup supplementation at levels of 0.0%, 0.1%, 0.2%, and 0.3% in the diets of quail (*Coturnix coturnix japonica*) on performance parameters, including body weight gain (BWG), feed intake (FI), feed conversion ratio (FCR), hot and cold carcass weights and carcass yields; meat quality characteristics, including L*, a*, b* color values, pH, and water-holding capacity (WHC) of breast and thigh meat; and oxidative stress parameters, including total antioxidant status (TAS), total oxidant status (TOS), and oxidative stress index (OSI); as well as glutathione peroxidase (*GPx7*) gene expression. A total of 120 newly hatched quail chicks were used in the experiment. The birds were assigned according to a completely randomized design with 15 replicates per treatment group and 2 birds per replicate and were randomly distributed into 60 cage compartments. While cumulative body weight gain (CBWG) was not significantly affected during the other experimental weeks (*p* > 0.05), statistically significant differences among the groups were observed only in the second week (*p* < 0.05). Feed intake values were not significantly different among the groups throughout all weeks of the experiment (*p* > 0.05). However, feed conversion ratio (FCR) was significantly different among the groups during all weeks (*p* < 0.05), with the pomegranate syrup-supplemented groups generally showing improved (lower) values compared with the control group. The inclusion of pomegranate syrup in the diets did not produce significant differences among the groups in breast meat quality parameters, including color values (L*, a*, b*), pH, and water-holding capacity. The obtained L*, a*, b*, and pH values were within the reference ranges reported in the literature. Evaluation of oxidative stress parameters demonstrated that pomegranate syrup supplementation increased TAS values, while significantly reducing TOS and OSI values (*p* < 0.05). Furthermore, *GPx7* gene expression increased in the pomegranate syrup-supplemented groups compared with the control group, with the highest expression observed at the 0.1% supplementation level. In addition, toxicological evaluations have confirmed the safety of pomegranate preparations, further supporting their potential as feed additives.

## Simple Summary

This study examined the effects of adding pomegranate syrup (0.0%, 0.1%, 0.2%, and 0.3%) to the diets of Japanese quail on growth performance, meat quality, and antioxidant status. A total of 120 quails were used and divided into four groups. The results showed that feed intake and meat quality traits were not affected by supplementation. However, feed efficiency was significantly changed. Pomegranate syrup improved antioxidant status by increasing total antioxidant levels and reducing oxidative stress markers. It also increased the expression of the antioxidant-related *GPx7* gene, especially at the 0.1% level. Overall, pomegranate syrup may enhance antioxidant defense in quails without negatively affecting growth or meat quality.

## 1. Introduction

Antibiotics and growth-promoting factors have long been incorporated into poultry diets as feed additives in order to improve feed efficiency and enhance growth performance. By altering the pH of the small intestine, antibiotics inhibit the survival and proliferation of harmful microorganisms that produce toxic substances for poultry, thereby positively affecting both animal health and productivity [1]. However, the prolonged use of antibiotic growth promoters may lead to the development of cross-resistance in pathogenic microorganisms and result in residue accumulation in animal-derived products. Consequently, this situation has led to the adoption of the “Antibiotic-Free Production” approach in the poultry industry [2]. Therefore, following the restriction and prohibition of antibiotics in animal nutrition, the search for alternative feed additives has intensified. In recent years, considerable attention has been focused on probiotics, prebiotics, organic acids, plant essential oils and extracts with antimicrobial and antioxidant properties, exogenous enzymes that support digestion, and toxin binders due to their beneficial effects on animal health [3,4].

Japanese quail (*Coturnix coturnix japonica*) are widely used in poultry research due to their rapid growth rate and intensive production characteristics. However, intensive production conditions, high metabolic activity, and environmental stressors can increase the production of reactive oxygen species, leading to oxidative stress. Oxidative stress occurs as a result of an imbalance between free radicals and the antioxidant defense system, and it may negatively affect growth performance, immune response, and meat quality. Therefore, antioxidant enzymes such as glutathione peroxidase (*GPx*) are considered important biomarkers in evaluating physiological responses to dietary interventions [5,6].

Pomegranate (*Punica granatum* L., Punicaceae) is one of the oldest known fruits. In addition to being consumed fresh, pomegranate can be processed into various products such as juice, juice concentrate, jam, and wine, and it is also widely used as a flavoring and coloring agent in foods [7]. In Türkiye, particularly in the Aegean, Mediterranean, and Southeastern Anatolia regions, pomegranate juice obtained by pressing the fruit is clarified and concentrated according to appropriate processing techniques to produce pomegranate syrup, which is commonly used to enhance the flavor of foods [8]. In recent years, increasing attention has been paid to the potential use of pomegranate juice by-products as natural antioxidants. Owing to their rich polyphenolic compound content, pomegranate by-products possess strong natural antioxidant properties, as demonstrated by both in vitro [9,10,11] and in vivo studies [12]. Furthermore, the potential health benefits of pomegranate, particularly in the prevention and treatment of cancer and cardiovascular diseases, have attracted considerable scientific interest. This has increased the importance of pomegranate as both a nutritional and medicinal product, while toxicological evaluations have also emphasized the safe use of pomegranate juice, extracts, and related preparations [13]. Despite its high sugar content, the beneficial effects of pomegranate syrup are mainly associated with its polyphenolic compounds, which enhance antioxidant defense mechanisms and reduce oxidative stress. Low dietary inclusion levels may also minimize potential negative metabolic effects of its carbohydrate content. However, limited information is available regarding the effects of pomegranate syrup supplementation on antioxidant status and *GPx* gene expression in Japanese quail (*Coturnix coturnix japonica*). In particular, studies investigating the combined effects of a sugar-rich polyphenol source on oxidative stress-related gene expression in poultry remain scarce [14].

This study was conducted to determine the effects of dietary supplementation of pomegranate syrup at levels of 0.0%, 0.1%, 0.2%, and 0.3% on growth performance, meat quality parameters, slaughter characteristics, oxidative stress parameters, and *GPx7* gene expression in Japanese quail (*Coturnix coturnix japonica*).

## 2. Materials and Methods

### 2.1. Animals and Diets

The study was conducted at the Poultry Unit of the Research and Application Farm of the Faculty of Veterinary Medicine, Harran University. The experiment was carried out in a multi-tiered battery cage system consisting of four levels, each equipped with independent rails and bulb lighting for illumination. The ambient temperature of the experimental room was regulated using a thermostat-controlled radiator and continuously monitored with a thermometer. The room was ventilated through a fan system connected to the external environment via wall openings. All animals were provided with feed and water ad libitum throughout the experiment. A total of 120 mixed-sex Japanese quail (*Coturnix coturnix japonica*) chicks at 10 days of age were used in the study. The birds were randomly allocated into 60 cage units according to a completely randomized design, with 15 replicates per group and 2 birds per replicate. Accordingly, the experiment consisted of one control group and three treatment groups. The quails were housed individually in cages measuring 30 × 20 × 20 cm (length × height × width), with one two birds per compartment. The environmental temperature was maintained at 21 °C throughout the experiment. A lighting program of 23 h light and 1 h darkness was applied using natural daylight and artificial lighting. The lighting program (23L:1D) was maintained at approximately 30 lux at bird level throughout the experiment. The nutrient composition of the experimental diets was formulated according to NRC [15] recommendations (Table 1). The diets were prepared with pomegranate syrup supplementation at levels of 0.0% (Group I), 0.1% (Group II), 0.2% (Group III), and 0.3% (Group IV). All diets were formulated to be isocaloric and isonitrogenous. The experiment lasted for 4 weeks. During diet preparation, pomegranate syrup was first thoroughly mixed with a portion of feed equivalent to five times its own weight. The mixture was then gradually incorporated into the remaining feed in a large container to ensure a homogeneous distribution.

The energy and nutrient composition of the pomegranate syrup used in the study per 100 g was as follows: 294 kcal energy, 72 g carbohydrates, 38 g sugars, 1 g protein, and <0.5 g salt.

The ingredient composition, nutrient contents (%), and metabolizable energy values (kcal/kg) of the experimental diets are presented in Table 1 below.

### 2.2. Productive Performance and Carcass Traits

In the present study, weekly body weights (BW) of the birds were recorded for 4 weeks using an electronic balance with a precision of 0.01 g. The birds were individually weighed at the beginning of the experiment and on days 7, 14, 21, and 28. The average daily body weight gain (ADG) was calculated by dividing the difference between two consecutive weighings by 7. On days 7, 14, 21, and 28 of the experiment, feed intake was determined by subtracting the remaining feed from the total feed offered to each bird. The average daily feed intake (ADFI) was calculated by dividing the feed consumption by the number of days (7 days). Feed conversion ratio (FCR) was calculated by dividing feed intake by body weight gain over each weighing interval. At the end of the experiment, slaughter procedures were carried out in a poultry processing facility by an experienced slaughter team. All procedures were performed under standardized conditions to ensure consistency and accuracy of measurements. Appropriate equipment, including a quail slaughter stand, scalding and defeathering equipment, evisceration and processing tables, a calibrated weighing balance, and an organ weighing platform, was used during the process. Prior to slaughter, birds were fasted overnight with free access to water. Quails were humanely slaughtered in accordance with animal welfare principles and standard processing protocols, ensuring minimal pain and stress. Slaughter was performed by decapitation following standard poultry processing guidelines. Following slaughter, carcasses were processed, and organ and carcass weights were recorded immediately using calibrated instruments. Feed was withdrawn overnight prior to slaughter. After slaughter, heart, liver, gizzard, and spleen weights were recorded for determination of organ and carcass traits. Following evisceration, carcasses were weighed to determine hot carcass weight. Subsequently, carcasses were chilled at +4 °C for 24 h and reweighed to obtain cold carcass weight. Carcass dissection was performed according to Genchev and Mihaylov [16]. Carcass yield was calculated as the ratio of carcass weight to pre-slaughter live weight. Wing, back-neck, thigh, and breast meat weights were individually recorded and expressed as a percentage of live body weight prior to slaughter.

### 2.3. Meat Quality Parameters

Meat quality parameters, including color and water-holding capacity (WHC), were determined in breast muscle samples. Breast meat color measurements, including lightness (L*), redness (a*), and yellowness (b*), were taken at three different points at 1 h and 24 h postmortem, and mean values were recorded. Color measurements were performed using a Lovibond RT Series SP60 colorimeter. Meat pH was measured using a Testo 205 pH meter. Water-holding capacity (WHC) of breast meat samples was determined according to Wardlaw et al. [17]. Approximately 8 g of breast meat was minced into pieces of approximately 0.25 cm^3^ and mixed with 12 mL of 0.6 M sodium chloride solution. The samples were centrifuged at 10,000 rpm for 15 min at +4 °C. The supernatant volume was measured, and WHC was expressed as a percentage.

### 2.4. Oxidative Stress Parameters

In serum samples, total antioxidant status (TAS) and total oxidant status (TOS) were determined using commercial kits (Rel Assay Diagnostics, Turkey) according to the manufacturer’s instructions. TAS measurement is based on the decolorization of the ABTS radical and was read spectrophotometrically at 660 nm; results were expressed as mmol Trolox equivalent/L. TOS analysis is based on the oxidation of the ferrous ion–o-dianisidine complex in an acidic medium and was measured at 530 nm; results were reported as μmol H_2_O_2_ equivalent/L. The oxidative stress index (OSI) was calculated as the ratio of TOS to TAS. At the end of the experimental period, liver tissue samples were collected immediately after slaughter, snap-frozen in liquid nitrogen, and stored at −80 °C until analysis.

### 2.5. Gene Expression Analysis

The expression levels of the target gene *GPX7* and the housekeeping gene *β-actin* were determined in liver tissue using real-time quantitative PCR (RT-qPCR). Liver tissue samples were homogenized using sterile marble beads under appropriate laboratory conditions. Total RNA was extracted from the homogenized tissues using TRIzol reagent according to the manufacturer’s instructions. The RNA isolation procedure was performed following the protocol described by Ndunguru et al. [18]. The concentration and purity of the isolated RNA were determined using a NanoDrop spectrophotometer.

Complementary DNA (cDNA) was synthesized from the isolated RNA using the Transcriptor High Fidelity cDNA Synthesis Kit (Roche, Germany) according to the manufacturer’s instructions. Real-time PCR reactions were prepared in a final volume of 25 µL containing 12.5 µL SYBR Green PCR Master Mix (Roche, Germany), 2 µL cDNA template, 2 µL each of forward and reverse primers (Table 1), and nuclease-free ddH_2_O. Amplification was performed using a Roche LightCycler 96 Real-Time PCR System with an initial denaturation at 95 °C for 10 min, followed by 40 cycles of denaturation at 95 °C for 15 s, annealing at 60 °C for 30 s, and extension at 72 °C for 30 s. *GPX7* (forward: 5′-TTGTAAACATCAGGGGCAAA-3′, reverse: 5′-TGGGCCAAGATCTTTCTGTAA-3′) and *β-actin* (forward: 5′-ACCCCAAAGCCAACAGA-3′, reverse: 5′-CCAGAGTCCATCACAATACC-3′) primers were used for RT-qPCR analysis as described by Bastos et al. [19].

### 2.6. Statistical Analyses

In this study, one-way analysis of variance (ANOVA) was used to determine whether there were statistically significant differences among the groups in terms of growth performance, carcass traits, and meat quality parameters. For growth performance data, the cage was considered the experimental unit. Since two birds were housed in each replicate cage, the mean values of birds within each cage were used for statistical comparisons, resulting in 15 experimental units per group. Individual birds were not considered as independent experimental units for performance parameters. For carcass and meat quality analyses, individual birds were considered as the sampling units. Prior to statistical analysis, data were checked for normality using the Shapiro–Wilk test and for homogeneity of variances using the Levene test. Statistical analyses were performed using IBM SPSS Statistics for Windows, Version 25.0 (IBM Corp., Armonk, NY, USA). When significant differences were detected among the groups, Duncan’s multiple range test was applied for post hoc comparisons. The level of statistical significance was set at *p* < 0.05 [20].

## 3. Results and Discussion

The effects of different levels of pomegranate syrup supplementation in quail diets on growth performance parameters are presented in Table 2. As shown in Table 2, no statistically significant differences among the groups were observed in terms of average daily body weight gain (ADG) during the 1st, 3rd, and 4th weeks (*p* > 0.05), whereas significant differences were detected among the treatment groups in the 2nd week (*p* < 0.05). Regarding feed intake values, no statistically significant differences were found among the groups throughout the entire 4-week experimental period (*p* > 0.05). However, feed conversion ratio (FCR) was significantly affected by dietary treatments in all weeks of the experiment (*p* < 0.05).

When the results regarding daily body weight gain were evaluated, it was observed that the supplementation of 0.0%, 0.1%, 0.2%, and 0.3% pomegranate syrup in the diets had a significant effect only among the groups in the second week (*p* < 0.05), whereas changes in body weight gain in the other weeks were not statistically significant (*p* > 0.05). In week 2, the 0.3% group had higher ADG than the 0.2% group, while control and 0.1% groups were intermediate (*p* = 0.043; Table 2). Deveci and Şengül [21] investigated the effects of dietary supplementation of different levels of pomegranate (*Thymbra spicata* L. var. *spicata*) seed oil (0%, 0.5%, 1%, 1.5%, and 2%) on growth performance and carcass characteristics in quail. In their study, Japanese quail chicks were used and the birds were subjected to a 42-day feeding trial. The authors reported that differences in mean values of body weight, feed intake, feed conversion ratio, carcass weight, and carcass yield among the control and treatment groups were not statistically significant. Similarly, Selçuk and Şengül [22] supplemented quail diets with two different levels (0.5 and 1 g/kg) of orange peel oil and pomegranate seed oil. While no significant differences were observed between the control and treatment groups in initial body weights, statistically significant differences in final body weights were reported at the end of the experiment. According to their findings, supplementation with 1 g/kg orange peel oil and 0.5 g/kg pomegranate seed oil had a significant effect on body weight (*p* < 0.05), whereas changes in the other groups were not statistically significant.

Abdel-Wahab and Mosad [23] reported that dietary supplementation of pomegranate peel powder at different levels (0.5–1.5%) significantly increased body weight in quail. In contrast, Saki et al. [24] stated that inclusion of pomegranate seed meal at levels of 5–15% in laying hen diets had no effect on body weight gain. Similarly, Abbas et al. [25] reported that the inclusion of pomegranate pomace powder at levels of 2.5–7.5% as a replacement for corn in quail diets did not significantly affect body weight.

When weekly feed intake values of control and treatment groups were evaluated, no statistically significant differences were observed among the groups throughout the 4-week experimental period (*p* > 0.05). In agreement with these findings, Yassein et al. [26] reported that dietary supplementation of pomegranate peel powder at levels of 10 and 15 g/kg significantly reduced feed intake in quail aged 12–20 weeks.

In the present study, when the mean feed conversion ratio (FCR) values of quail in the treatment groups were evaluated, statistically significant differences were observed among all weeks (*p* < 0.05).

Abdel-Wahab and Mosad [23] reported that supplementation of pomegranate peel powder at different levels (0.5–1.5%) significantly improved feed efficiency in quail. In contrast, Saki et al. [24] stated that inclusion of pomegranate seed meal at levels of 5–15% in laying hen diets did not affect feed conversion ratio.

When the results obtained in the present study regarding growth performance are compared with those reported in previous studies, both similar and differing findings can be observed. Such variability in results is commonly reported in studies involving plant extracts in poultry nutrition. These discrepancies may be attributed to several factors, including diet composition, feed intake levels, environmental conditions where the plants are grown, harvest time, extraction methods and storage conditions of the extracts, as well as differences in inclusion levels used in the diets [27,28].

The effects of dietary supplementation of different levels of pomegranate syrup on slaughter characteristics and internal organ weights in quail are presented in Table 3. As shown in Table 3, no statistically significant differences were observed among the groups (*p* > 0.05).

The effects of dietary supplementation of different levels of pomegranate syrup on breast meat quality parameters, including color (L*, a*, b*), pH, and water-holding capacity (WHC), are presented in Table 4. As shown in Table 4, the inclusion of pomegranate syrup in the diet did not result in any significant differences among the groups in terms of pH, WHC, or breast meat color parameters (L*, a*, and b*).

The obtained L*, a*, b*, and pH values were within the reference ranges reported in the literature [29,30,31].

Genchev et al. [32] reported that in Japanese quail, breast meat color coordinates in the lightness (L*) spectrum ranged from 40.6 to 56.1, redness/greenness (a*) values ranged from 5.1 to 14.4, and yellowness/blueness (b*) values ranged from 7.9 to 13.4. In another study, Betti et al. [29] evaluated L*, a*, and b* values in broiler breast meat and reported that L* values ranged between 40 and 66, a* values between 0 and −13, and b* values between −3 and 12 at the end of the experiment.

Sarmiento-Garcia et al. [33] investigated the effects of dietary supplementation of pomegranate seed oil at levels of 100 and 200 mg/kg on breast and thigh meat quality in broiler quail. They reported that the highest L* and b* values were observed in the breast meat of birds receiving 100 mg/kg pomegranate seed oil, while in thigh meat, the highest values were recorded in the group receiving 200 mg/kg supplementation.

In the present study, the lack of significant differences in pH, WHC, and breast meat color parameters (L*, a*, and b*) among the treatment groups may be attributed to the low inclusion level of pomegranate syrup in the diets.

The effects of dietary supplementation of different levels of pomegranate syrup on total antioxidant status (TAS), total oxidant status (TOS), and oxidative stress index (OSI) are presented in Table 5.

As shown in Table 5, pomegranate syrup supplementation significantly increased total antioxidant status (TAS) compared with the control group (*p* < 0.05). The highest TAS value was observed in the group supplemented with 0.2% pomegranate syrup, and all pomegranate-supplemented groups exhibited higher TAS values than the control group in a similar manner.

Regarding total oxidant status (TOS), statistically significant differences were detected among the groups (*p* < 0.05), with the lowest TOS value recorded in the 0.3% pomegranate syrup group. The control group showed higher TOS values compared with all pomegranate-supplemented groups.

When oxidative stress index (OSI) was evaluated, pomegranate syrup supplementation was found to significantly reduce OSI levels (*p* < 0.05). The control group had the highest OSI value, whereas all supplemented groups exhibited lower and similar OSI values.

These findings indicate that pomegranate syrup may exert a protective effect against oxidative stress. It has been reported that phenolic compounds, flavonoids, and anthocyanins in pomegranate suppress free radical formation, reduce lipid peroxidation, and enhance the antioxidant defense system [34]. Furthermore, several studies have demonstrated that dietary inclusion of pomegranate by-products in poultry diets improves antioxidant capacity and alleviates oxidative stress parameters [35,36]. Therefore, the observed effects in the present study may be attributed to the biologically active phenolic compounds in pomegranate, which may modulate oxidative stress mechanisms.

**Figure 1:**
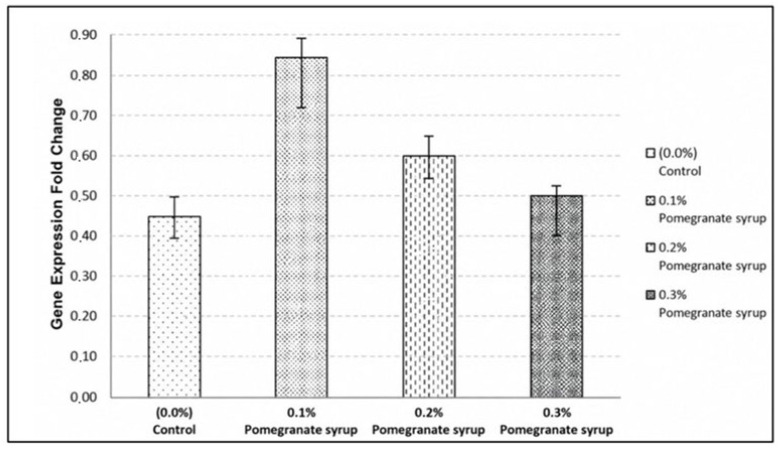
Comparison of *GPx7* gene expression levels among the experimental groups.

The effects of dietary supplementation of different levels of pomegranate syrup on *GPx7* gene expression in quail are presented in Figure 1.

As shown in Figure 1, dietary supplementation of pomegranate syrup increased *GPx7* gene expression compared with the control group. The highest *GPx7* gene expression was observed in the 0.1% group, followed by the 0.2% group, whereas the 0.3% group exhibited values close to those of the control group. In contrast, *GPx7* gene expression in the 0.3% group decreased to levels close to those of the control group. The lower *GPx7* gene expression observed in the 0.3% pomegranate syrup group may indicate that the beneficial effects of pomegranate polyphenols are dose-dependent. At higher concentrations, some polyphenolic compounds may exert pro-oxidant activity under certain physiological conditions, which can suppress endogenous antioxidant defense mechanisms through negative feedback regulation. Similar biphasic (hormetic) responses of dietary polyphenols have been reported in the literature, where low doses stimulate antioxidant defenses, whereas excessive doses may attenuate these responses [37,38].

This response may be associated with the phenolic compounds and natural antioxidant constituents present in pomegranate syrup, which may enhance the antioxidant defense system and contribute to the upregulation of *GPx7* gene expression. It has been reported that pomegranate-derived polyphenols can increase antioxidant enzyme gene expression [39]. Similarly, it has been suggested that plant-derived antioxidants, when administered at appropriate levels, can stimulate cellular defense mechanisms and enhance antioxidant responses [35]. In addition, studies in poultry have reported that phytogenic feed additives may modulate the antioxidant defense system and increase the expression of antioxidant genes, including *GPx* [40].

These findings indicate that plant-derived compounds can modulate the antioxidant defense system not only at the enzymatic activity level but also at the gene expression level. In the present study, the more pronounced increase in *GPx7* gene expression observed at the 0.1% supplementation level suggests that low levels of pomegranate syrup may stimulate cellular defense mechanisms and provide a protective effect against oxidative stress.

## 4. Conclusions

According to the results of the present study, pomegranate syrup supplementation had no marked negative effects on growth performance, carcass characteristics, internal organ weights, or breast meat quality parameters in quail. In addition, meat quality traits such as color coordinates (L*, a*, b*), pH, and water-holding capacity were found to be within the reference ranges. Regarding oxidative stress parameters, pomegranate syrup supplementation increased total antioxidant status (TAS) while reducing total oxidant status (TOS) and oxidative stress index (OSI). Similarly, *GPx7* gene expression was also upregulated with pomegranate syrup supplementation, with the highest expression level observed in the 0.1% supplementation group. However, a reduction in *GPx7* expression at the highest inclusion level (0.3%) was observed, indicating a possible non-linear dose-dependent response. In conclusion, dietary supplementation of pomegranate syrup in quail diets may positively modulate antioxidant defense mechanisms and oxidative stress-related biomarkers. The findings suggest that pomegranate syrup has potential as a natural feed additive; however, further studies with larger sample sizes and longer experimental periods are required to confirm these effects. When the results are evaluated together, it is observed that 0.1% pomegranate syrup supplementation produced the highest increase in *GPx7* gene expression and, by increasing TAS while decreasing TOS and OSI, provided the most balanced and most favorable biological response in terms of oxidative stress status.

## Figures and Tables

**Table 1:** Ingredient composition, chemical composition, and calculated nutrient levels of the experimental diets.

Ingredients (%)	Control	0.1% Pomegranate Syrup	0.2% Pomegranate Syrup	0.3% Pomegranate Syrup
Wheat	3.41	3.34	3.34	2.63
Corn	53.82	53.79	53.69	54.30
Fish meal	0.20	0.20	0.20	0.20
Soybean meal (48% CP)	39.00	39.00	39.00	39.00
Vegetable oil	0.28	0.28	0.28	0.28
Pomegranate syrup	-	0.1	0.2	0.3
Limestone	1.36	1.36	1.35	1.35
Dicalcium phosphate	0.62	0.62	0.63	0.63
DL-Methionine	0.40	0.40	0.40	0.40
L-Threonine	0.13	0.13	0.13	0.13
Salt	0.38	0.38	0.38	0.38
Vitamin–mineral premix	0.40	0.40	0.40	0.40
**Total**	**100**	**100**	**100**	**100**
**Analyzed values (%)**				
Dry matter	89.7	89.7	89.7	89.8
Crude protein	24.0	24.0	24.0	24.0
Crude fat	2.10	2.10	2.10	2.09
Crude fiber	2.81	2.81	2.80	2.80
Crude ash	5.76	5.76	5.76	5.75
**Calculated nutrient levels**				
Metabolizable energy (kcal/kg)	2904	2901	2897	2897
Calcium (Ca) (%)	0.80	0.80	0.80	0.80
Available phosphorus (%)	0.30	0.30	0.30	0.30
Methionine + cystine (%)	1.20	1.20	1.20	1.20
Lysine (%)	1.32	1.32	1.32	1.32

Vitamin and mineral contents per kilogram of the experimental diets were as follows: vitamin A, 12,000 IU; vitamin D_3_, 5000 IU; vitamin E, 50 mg; vitamin K_3_, 4 mg; vitamin B_1_, 3 mg; vitamin B_2_, 6 mg; niacin, 40 mg; calcium D-pantothenate, 15 mg; vitamin B_6_, 5 mg; vitamin B_12_, 0.03 mg; folic acid, 1 mg; biotin, 0.075 mg; choline chloride, 400 mg; vitamin C, 50 mg; and antioxidant, 10 mg. The mineral composition included manganese, 120 mg; iron, 40 mg; zinc, 110 mg; copper, 16 mg; cobalt, 0.005 mg; iodine, 0.125 mg; and selenium, 0.003 mg.

**Table 2:** Effects of dietary pomegranate syrup supplementation on growth performance parameters (ADG, FI, and FCR; mean ± SEM).

	Groups	Week 1	Week 2	Week 3	Week 4
**ADG**	Control	3.98 ± 0.12	5.30 ± 0.08 ^ab^	5.36 ± 0.27	3.42 ± 0.50
	0.1% Pomegranate syrup	4.38 ± 0.19	5.48 ± 0.18 ^ab^	5.49 ± 0.54	3.41 ± 0.63
	0.2% Pomegranate syrup	3.74 ± 0.16	5.15 ± 0.18 ^b^	5.40 ± 0.36	4.06 ± 0.51
	0.3% Pomegranate syrup	4.61 ± 0.51	5.73 ± 0.14 ^a^	5.33 ± 0.42	3.62 ± 0.47
	*p*-value	0.135	0.043	0.994	0.794
**FI**	Control	15.87 ± 0.72	22.76 ± 0.40	25.32 ± 0.89	26.91 ± 1.35
	0.1% Pomegranate syrup	16.78 ± 0.37	24.14 ± 0.63	27.04 ± 0.77	27.48 ± 1.31
	0.2% Pomegranate syrup	15.60 ± 0.34	21.98 ± 0.67	25.84 ± 0.44	27.61 ± 0.69
	0.3% Pomegranate syrup	16.28 ± 0.49	23.21 ± 0.42	26.63 ± 0.60	27.08 ± 0.46
	*p*-value	0.392	0.05	0.305	0.956
**FCR**	Control	3.98 ± 0.006 ^b^	4.29 ± 0.004 ^b^	4.72 ± 0.004 ^d^	7.86 ± 0.017 ^b^
	0.1% Pomegranate syrup	3.83 ± 0.006 ^c^	4.40 ± 0.004 ^a^	4.92 ± 0.004 ^b^	8.05 ± 0.017 ^a^
	0.2% Pomegranate syrup	4.17 ± 0.004 ^a^	4.26 ± 0.006 ^c^	4.77 ± 0.006 ^c^	6.80 ± 0.081 ^d^
	0.3% Pomegranate syrup	3.53 ± 0.004 ^d^	4.05 ± 0.004 ^d^	4.99 ± 0.015 ^a^	7.48 ± 0.009 ^c^
	*p*-value	0.000	0.000	0.000	0.000

ADG: Average Daily Weight Gain; FI: Feed intake; FCR: Feed conversion ratio. *p*-value interpretation: Significant (*p* < 0.05); Not significant (*p* > 0.05). a, b, c, d: Means within the same row with different superscripts differ significantly (*p* < 0.05), SEM: Standard error of the mean.

**Table 3:** Effects of dietary pomegranate syrup supplementation on slaughter characteristics and internal organ weights (Mean ± SEM).

	Control	0.1% Pomegranate Syrup	0.2% Pomegranate Syrup	0.3% Pomegranate Syrup	*p*-Value
Slaughter weight	186.14 ± 6.45	189.88 ± 4.93	178.58 ± 4.44	186.02 ± 4.92	0.525
Carcass weight	116.77 ± 2.31	118.41 ± 2.09	110.26 ± 3.15	120.01 ± 3.66	0.105
Carcass yield (dressing percentage)	63.26 ± 1.35	62.76 ± 1.57	61.97 ± 1.70	64.64 ± 1.41	0.655
Breast	24.82 ± 0.47	25.09 ± 0.67	24.45 ± 0.56	24.40 ± 0.66	0.829
Thigh	14.09 ± 0.37	14.37 ± 0.40	14.09 ± 0.39	14.64 ± 0.28	0.681
Back + neck	20.29 ± 0.83	19.14 ± 0.72	18.99 ± 0.91	21.50 ± 0.11	0.188
Wing	3.83 ± 0.11	3.91 ± 0.14	4.06 ± 0.13	4.10 ± 0.14	0.423
Liver	1.96 ± 0.18	1.82 ± 0.14	2.01 ± 0.16	1.17 ± 0.13	0.545
Gizzard	2.13 ± 0.06	2.02 ± 0.07	2.00 ± 0.06	2.09 ± 0.06	0.460
Heart	1.06 ± 0.04	1.04 ± 0.06	1.05 ± 0.04	1.17 ± 0.05	0.160

*p*-value: Significant (*p* <0.05); Not significant (*p* > 0.05). SEM: Standard error of the mean.

**Table 4:** Effects of dietary pomegranate syrup supplementation on breast meat quality parameters, including color (L, a, b*), pH, and WHC (Mean ± SEM).

	Control	0.1% Pomegranate Syrup	0.2% Pomegranate Syrup	0.3% Pomegranate Syrup	*p*-Value
L*	46.98 ± 0.66	46.25 ± 0.86	46.98 ± 0.46	46.51 ± 0.98	0.884
a*	12.48 ± 0.48	11.01 ± 0.68	10.78 ± 0.56	11.78 ± 0.33	0.097
b*	11.55 ± 0.39	10.29 ± 0.49	10.77 ± 0.31	9.97 ± 0.52	0.064
**WHC**	5.46 ± 0.08	5.45 ± 0.15	5.52 ± 0.15	5.52 ± 0.16	0.973
**pH**	5.92 ± 0.03	5.91 ± 0.023	5.96 ± 0.021	6.15 ± 0.14	0.087

a*: Redness coordinate; b*: Yellowness coordinate; L*: Lightness. WHC: Water-holding capacity. (*p* < 0.05): Differences among means within the same column with different superscripts are significant. *p*-value: Significant (*p* < 0.05); Not significant (*p* > 0.05); SEM: Standard error of the mean.

**Table 5:** Effects of dietary pomegranate syrup supplementation on oxidative stress parameters (Mean ± SEM).

Groups	TAS	TOS	OSI
**Control**	1.47 ± 0.09 ^b^	14.19 ± 0.47 ^a^	0.99 ± 0.04 ^a^
**0.1% Pomegranate Syrup**	2.11 ± 0.15 ^a^	13.61 ± 1.95 ^ab^	0.66 ± 0.09 ^b^
**0.2% Pomegranate Syrup**	2.20 ± 0.13 ^a^	11.62 ± 0.45 ^ab^	0.55 ± 0.05 ^b^
**0.3% Pomegranate Syrup**	2.07 ± 0.17 ^ba^	10.68 ± 0.34 ^b^	0.55 ± 0.05 ^b^
***p*** **-value**	0.031	0.000	0.000

TAS: Total Antioxidant Status; TOS: Total Oxidant Status; OSI: Oxidative Stress Index; (*p* < 0.05): Differences among means within the same column with different superscripts are significant; SEM: Standard error of the mean; *p*-value: Significant (*p* < 0.05); Not significant (*p* > 0.05).

## Data Availability

The original contributions presented in this study are included in the article. Further inquiries can be directed to the corresponding author.

## References

[1] Sarıca Ş. (2011). Nar suyu yan ürünlerinin hayvan beslemede kullanım olanakları. Gaziosmanpaşa Üniv. Ziraat Fak. Derg..

[2] Olmez M., Karadağoğlu O., Berberoğlu T.M., Sarı E.K., Aras S.Y., Yılmaz B., Şahin T. (2023). Etlik bıldırcın (*Coturnix coturnix japonica*) rasyonlarına prebiyotik kombinasyonu ilavesinin büyüme performansı ve duodenum histolojisi üzerindeki etkileri. Kadirli Uygulamalı Bilim. Fak. Derg..

[3] Gürsoy E. (2021). Bitkisel ekstraktların hayvan beslemede kullanımı. Kadirli Uygulamalı Bilim. Fak. Derg..

[4] Kheravii S.K., Swick R.A., Choct M., Wu S.B. (2016). The changes of short-chain fatty acids and cecal bacteria in response to a lignocellulose supplementation in wheat or corn based diet. Poult. Sci..

[5] Sies H. (1997). Oxidative stress: Oxidants and antioxidants. Exp. Physiol..

[6] Surai P.F. (2002). Natural Antioxidants in Avian Nutrition and Reproduction.

[7] Marti N., Perez-Vicente A., Garcia-Viguera C. (2001). Influence of storage temperature and ascorbic acid addition on pomegranate juice. J. Sci. Food Agric..

[8] Vardin H., Abbasoğlu M. Nar ekşisi ve narın diğer değerlendirme olanakları. Proceedings of the Geleneksel Gıdalar Sempozyumu.

[9] Gil M.I., Tomas-Barberan F.A., Hess-Pierce B., Holcroft D.M., Kader A.A. (2000). Antioxidant activity of pomegranate juice and its relationship with phenolic composition and processing. J. Agric. Food Chem..

[10] Singh R.P., Chidambara M.K.N., Jayaprakasha G.K. (2002). Studies on antioxidant activity of pomegranate peel and seed extracts. J. Agric. Food Chem..

[11] Li Y., Guo C., Yang J., Wei J., Xu J., Cheng S. (2006). Evaluation of antioxidant properties of pomegranate peel extract. Food Chem..

[12] Shabtay A., Eitam H., Tadmor Y., Orlov A., Meir A., Weinberg P., Weinberg Z.G., Hen Y., Brosh A., Izhaki I. (2008). Nutritive and antioxidative potential of pomegranate by-product. J. Agric. Food Chem..

[13] Kumar N., Kumar N.S. (2018). Functional properties of pomegranate (*Punica granatum* L.). Pharma Innov. J..

[14] Hashemipour H., Kermanshahi H., Golian A., Veldkamp T. (2013). Effect of thymol and carvacrol feed supplementation on performance, antioxidant enzyme activities, fatty acid composition, digestive enzyme activities, and immune response in broiler chickens. Poult. Sci..

[15] NRC (1994). Nutrient Requirements of Poultry.

[16] Genchev A., Mihaylov R. (2008). Slaughter analysis protocol in Japanese quails. Trakia J. Sci..

[17] Wardlaw F.B., McCaskill L.H., Acton J.C. (1973). Effect of postmortem muscle changes on poultry meat loaf properties. J. Food Sci..

[18] Ndunguru S.F., Reda G.K., Csernus B., Knop R., Gulyás G., Szabó C., Lendvai Á.Z. (2024). Embryonic methionine triggers post-natal developmental programming in Japanese quail. J. Comp. Physiol. B.

[19] Bastos M.S., Del Vesco A.P., Santana T.P., Santos T.S., de Oliveira Junior G.M., Fernandes R.P.M., Silva L.T., Gasparino E. (2017). The role of cinnamon as a modulator of the expression of genes related to antioxidant activity and lipid metabolism of laying quails. PLoS ONE.

[20] IBM Corp (2017). IBM SPSS Statistics for Windows, Version 25.0.

[21] Deveci M., Şengül T. (2022). Bıldırcın Diyetlerine Farklı Düzeylerde Nar Çekirdeği Yağı İlavesinin Besi Performansı ve Karkas Özelliklerine Etkisi. Türk Tarım Doğa Bilim. Derg..

[22] Selçuk Ş., Şengül T. (2021). Portakal kabuğu yağı ve nar çekirdeği yağı ile zenginleştirilen diyetlerin bıldırcınların verim performansı, yumurta kalitesi ve bazı kan parametreleri üzerine etkileri. Türk Tarım Doğa Bilim. Derg..

[23] Abdel-Wahab A.A., Mosad A.S. (2018). Effect of adding pomegranate peels to growing Japanese quail diet on performance, blood and immunity parameters. Egypt. J. Nutr. Feed..

[24] Saki A.A., Rabet M., Zamani P., Yousefi A. (2014). The effects of different levels of pomegranate seed pulp with multi-enzyme on performance, egg quality and serum antioxidant in laying hens. Iran. J. Appl. Anim. Sci..

[25] Abbas R.J., Al-Salhie K.C.K., Al-Hummod S.K.M. (2017). The effect of using different levels of pomegranate (*Punica granatum*) peel powder on productive and physiological performance of Japanese quail (*Coturnix coturnix japonica*). Livest. Res. Rural Dev..

[26] Yassein D.M.M., Abdallah E.A., Ismail I.I., Faddle A.A. (2015). Effect of dietary supplementation of pomegranate peel powder and butylated hydroxy toluene on some productive, physiological and immunological parameters of Japanese quail. Egypt. J. Nutr. Feed..

[27] Brenes A., Roura E. (2010). Essential oils in poultry nutrition: Main effects and modes of action. Anim. Feed Sci. Technol..

[28] Hashemi S.R., Davoodi H. (2011). Herbal plants and their derivatives as growth and health promoters in animal nutrition. Vet. Res. Commun..

[29] Betti M., Fletcher D.L. (2005). The influence of extraction and precipitation pH on the dry matter yield of broiler dark meat. Poult. Sci..

[30] Boni I., Nurul H., Noryati I. (2010). Comparison of meat quality characteristics between young and spent quails. Int. Food Res. J..

[31] Genchev A., Mihaylova G., Ribarski S., Pavlov A., Kabakchiev M. (2008). Meat quality and composition in Japanese quails. Trakia J. Sci..

[32] Genchev A., Ribarski S., Zhelyazkov G. (2010). Physicochemical and technological properties of Japanese quail meat. Trakia J. Sci..

[33] Sarmiento-García A., Gökmen S.A., Sevim B., Olgun O. (2023). Performance and meat quality characteristics of male quails (*Coturnix coturnix japonica*) fed diets supplemented with pomegranate seed oil. Span. J. Agric. Res..

[34] Mertens-Talcott S.U., Jilma-Stohlawetz P., Rios J., Hingorani L., Derendorf H. (2006). Absorption, metabolism, and antioxidant effects of pomegranate (*Punica granatum* L.) polyphenols after ingestion of a standardized extract in healthy human volunteers. J. Agric. Food Chem..

[35] Ahmadipour B., Pat S., Abaszadeh S., Hassanpour H., Khajali F. (2021). Pomegranate peel as a phytogenic in broiler chickens: Influence upon antioxidant, lipogenesis and hypotensive response. Vet. Med. Sci..

[36] Ghasemi-Sadabadi M., Ebrahimnezhad Y., Maheri-Sis N., Ghalehkandi J.G., Shaddel-Teli A. (2021). Immune response and antioxidant status of broilers as influenced by oxidized vegetable oil and pomegranate peel. J. Anim. Sci. Technol..

[37] Galati G., O’Brien P.J. (2004). Potential toxicity of flavonoids and other dietary phenolics: Significance for their chemopreventive and anticancer properties. Free Radic. Biol. Med..

[38] Calabrese E.J., Mattson M.P., Calabrese V. (2010). Dose response biology: The case of resveratrol. Hum. Exp. Toxicol..

[39] Li X., Liu L., Pischetsrieder M. (2017). Pomegranate (*Punica granatum* L.) wine polyphenols affect Nrf2 activation and antioxidant enzyme expression in human neuroblastoma cells (SH-SY5Y). J. Funct. Foods.

[40] Griela E., Paraskeuas V., Mountzouris K.C. (2021). Effects of diet and phytogenic inclusion on the antioxidant capacity of the broiler chicken gut. Animals.

